# Navigation of Chemotactic Cells by Parallel Signaling to Pseudopod Persistence and Orientation

**DOI:** 10.1371/journal.pone.0006842

**Published:** 2009-08-31

**Authors:** Leonard Bosgraaf, Peter J. M. Van Haastert

**Affiliations:** Department of Cell Biochemistry, University of Groningen, Haren, The Netherlands; Massachusetts General Hospital/Harvard University, United States of America

## Abstract

The mechanism of chemotaxis is one of the most interesting issues in modern cell biology. Recent work shows that shallow chemoattractant gradients do not induce the generation of pseudopods, as has been predicted in many models. This poses the question of how else cells can steer towards chemoattractants. Here we use a new computational algorithm to analyze the extension of pseudopods by *Dictyostelium* cells. We show that a shallow gradient of cAMP induces a small bias in the direction of pseudopod extension, without significantly affecting parameters such as pseudopod frequency or size. Persistent movement, caused by alternating left/right splitting of existing pseudopodia, amplifies the effects of this bias by up to 5-fold. Known players in chemotactic pathways play contrasting parts in this mechanism; PLA2 and cGMP signal to the cytoskeleton to regulate the splitting process, while PI 3-kinase and soluble guanylyl cyclase mediate the directional bias. The coordinated regulation of pseudopod generation, orientation and persistence by multiple signaling pathways allows eukaryotic cells to detect extremely shallow gradients.

## Introduction

Chemotaxis plays essential roles in development, metastasis and finding bacteria during infection [1]–[3]. It is generally accepted that during chemotaxis positional cues induce a bias of pseudopod extension, by which cells move on average more often in the direction of the chemoattractant gradient than in other directions [1]. To understand the mechanisms by which cells navigate in a gradient of chemoattractant we have first investigated how cells extend pseudopodia in the absence of external cues, and then characterized the bias of size, direction or position of pseudopodia that is induced by the gradient. Cells in the absence of external cues do not move in random directions but exhibit a so-called correlated random walk [4]–[8]. This tendency to move in the same direction is called persistence. Cells with strong persistence make fewer turns, move for prolonged periods of time in the same direction, and thereby effectively penetrate into the surrounding. This suggests that persistence may have a major impact on how cells colonize a new environment. By increasing the persistence time, cells disperse better during food seeking [9], move longer distances during morphogenesis [10], [11] and may escape into the environment during metastasis [12], [13]. Chemotaxis may represent another field of cell biology where persistence could be critical, because cells moving without persistence need a chemotaxis bias for each new pseudopod, while cells moving persistently will accumulate directional accuracy at each subsequent pseudopod.

To investigate how pseudopod extension regulates cell movement we developed a computer algorithm that identifies the size, timing and direction of extending pseudopodia, as well as the local curvature of the cell boundary at the position where the pseudopodia emerge [14]. *Dictyostelium* cells, like neutrophils and many other amoeboid cells, can extend two types of pseudopodia [15]. New protrusions originate predominantly by splitting of an existing pseudopod. The cells may also extend pseudopodia from areas of the cell not previously active, which we describe as *de novo* pseudopodia (often referred to as “lateral pseudopodia” because they often appear at the side and in the rear of the cell). By analyzing the extension of ∼2000 pseudopodia by *Dictyostelium* cells in buffer we have shown that split pseudopodia are extended predominantly alternating left/right at a small angle leading to a nearly straight persistent path, while *de novo* pseudopodia are extended in nearly random directions. Therefore persistence is determined by the ratio of split/*de novo* pseudopodia [16]. Here we describe how pseudopodia are extended during chemotaxis of wild type and mutant *Dictyostelium* cells. We identify the mechanisms of four signaling pathways that cells use to bias pseudopod extension in the direction of a shallow gradient of the chemoattractant cAMP.

## Results

As described in the introduction, amoeboid cells in the absence of external cues exhibit persistence: they have a high probability to extend pseudopodia in a similar direction as previous pseudopodia. During chemotaxis cells also exhibit orientation: the gradient induces a bias in the average direction of movement towards cAMP. We first investigated how cells orient in a gradient, then analyzed the role of persistence, and finally measured orientation and persistence in signaling mutants and during natural chemotaxis.

### Bias of pseudopod extension by chemotactic gradients

**Figure 1:**
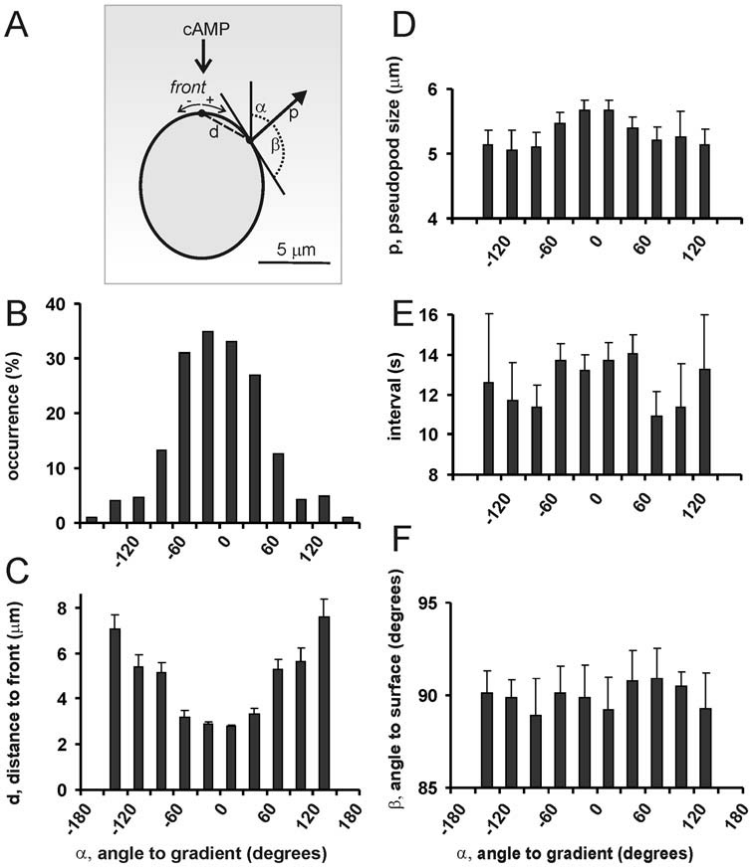
A cAMP gradient induces a bias of the position where pseudopodia emerge. Starved wild type cells were exposed to a cAMP gradient. A, the extension of 835 pseudopodia were recorded by the pseudopod algorithm, which also identifies the *front* of the cell as the position of the cell outline that is most nearby the cAMP source. The analysis contains information on the size of each pseudopod, the time interval between two pseudopodia, the angle *α* of the pseudopod relative to the gradient, the angle *β* of the pseudopod relative to the tangent to the cell surface where the pseudopod emerges, and the distance *d* between *front* and position where the pseudopod emerges. Data are means and SEM, with n the number of pseudopodia. Panel B shows the probability frequency distribution of pseudopodia with different directions relative to the gradient. The results of panels C-F reveal that the cAMP gradient does not bias the interval between pseudopodia (E), or the angle β relative to the surface (F). The gradient has a small effect on the size of the pseudopod (D, none of the bars is statistically significantly different from any other bar; however, the pool of all data with −30<α<+30 degrees [two central bars 5.68 +/− 2.00 µm, n = 350] and the pool of all data with α<−60 or α>+60 [three outer bars at each side 5.14 +/− 1.43 µm, n = 174] are statistically significant at P<0.01). The cAMP gradient strongly enhances the probability that pseudopodia emerge nearby the *front* (C). Therefore, pseudopodia emerging perpendicular to the surface of a spherical body at a short distance from the *front* must have a small angle α, and are automatically directed towards the gradient.

Wild type cells were exposed to a shallow gradient of cAMP (mean concentration is 650 nM, the spatial gradient is 0.7% across the cell). Movies were recorded, and with the computer algorithm Quimp3 data were collected for 835 pseudopodia extended by 28 cells. We measured the size, interval, direction *α* towards the gradient and direction *β* towards the membrane curvature (Fig. 1A). Obviously for cells moving towards cAMP, many pseudopodia are extended in the direction of the gradient (Fig. 1B). In the absence of spatial cues, pseudopodia are extended perpendicular to the surface of the cell [16]. The gradient induces a strong bias of the position where pseudopodia emerge, such that pseudopodia appear more likely at the side of the cell closer towards the gradient than at other sides of the cell (Fig. 1C). The sizes of pseudopodia that are extended in the direction of the gradient are slightly larger than pseudopodia extended in other directions (Fig. 1D; see legend for statistics). Furthermore, the time interval between the extension of pseudopodia is not affected by the gradient (Fig. 1E). Finnally, in the cAMP gradient as in buffer xxref, pseudopodia are extended still perpendicular to the local surface curvature, independent of where the pseudopodia emerged, suggesting that the pseudopodia are not bent towards the gradient (Fig. 1F).

The results of figure 1 imply that the direction of cell movement is mediated by pseudopodia that are extended at the side of the cell closest to the gradient. We investigated how this orientation is brought about: by the extension of pseudopodia at those sides and/or by selective retraction of pseudopodia at other sides. *Dictyostelium* cells in buffer or in a cAMP gradient occasionally (∼20% of time) extend two pseudopodia, of which one pseudopod is retraced, probably to retain polarity of the cell. In a cAMP gradient the retracted pseudopodia are oriented at 84 +/− 8 degrees relative to the gradient, while maintained pseudopodia are oriented significantly better at 37 +/− 3 degrees (mean and SEM, n = 50; see supplemental Fig. S1). Thus, whenever cells have multiple pseudopodia, selective retraction contributes to chemotaxis. However, the predominant way cells move is not by symmetric splitting a pseudopod into two and retracting one of them [15], but by splitting-off a pseudopod alternating to the right and left as an ice-skater [16].

**Figure 2:**
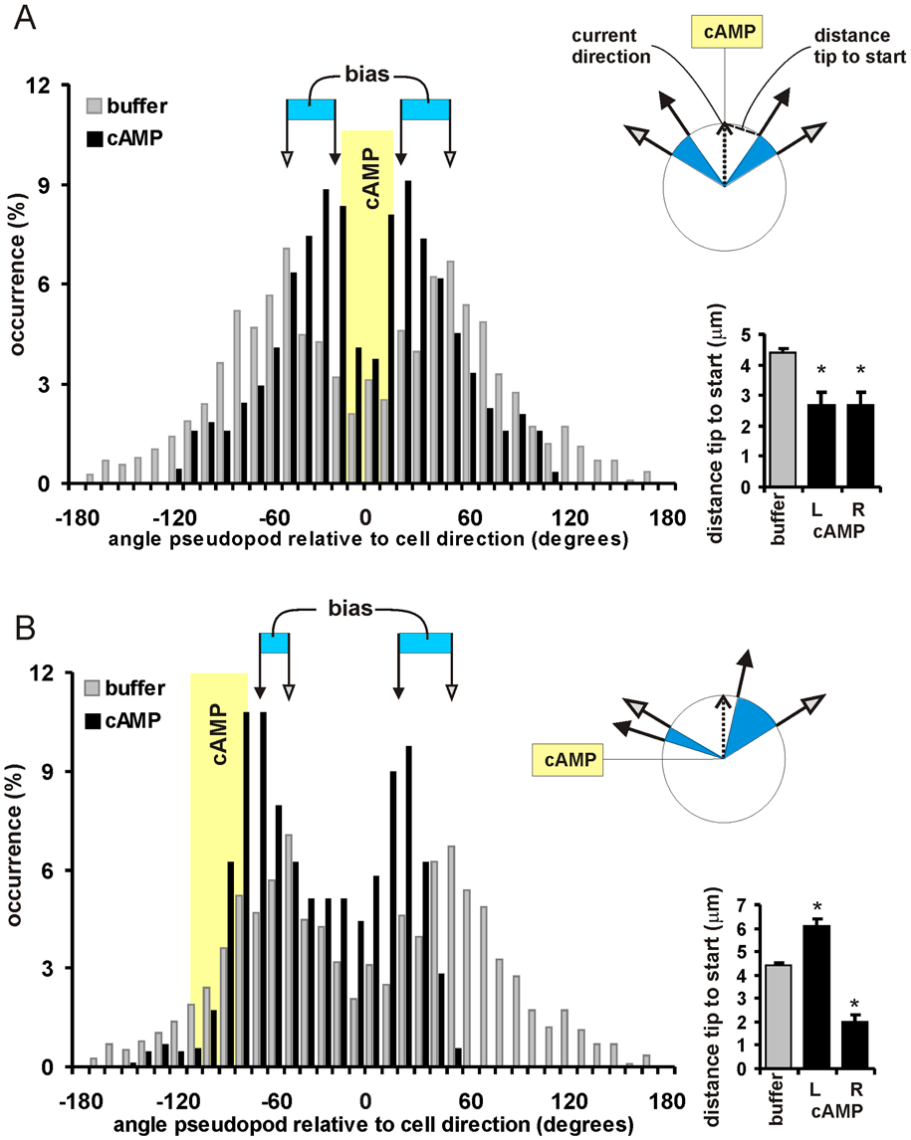
Orientation of *Dictyostelium* cells in shallow gradients. From a large data set of pseudopodia that are extended by freely moving cells in a cAMP gradient (see supplemental figure S2 for large data set), we selected those cells whose current direction of movement is either in the direction of the cAMP gradient (−20 to + 20 degrees), or at an angle of ∼90 degrees relative to the gradient (−70 to −110 degrees). The position of the cAMP gradient is shown by the yellow bar. The main figures show the histograms of the angles between current pseudopod and next pseudopod. In buffer this angle has a bi-symmetric distribution with 55 +/− 28 degrees to the left or right (grey bars; mean and SD, wrapped von Mises distribution). If the current direction is towards cAMP (panel A, solid bars), the distribution of angles is also bi-symmetric but at a smaller mean and smaller SD (30 +/− 18 degrees), leading to a bias towards cAMP (blue area). If the cAMP gradient is at an angle of ∼90 degrees to the left relative to the current direction (panel B), the next pseudopod exhibits an asymmetric bias towards cAMP with −70 +/− 23 degrees for the left pseudopod and 18 +/− 20 degrees for the right pseudopod. The inset bar graphs show the distance between the tip of the present pseudopod to the start of the next pseudopod; *, significantly different from buffer at P<0.01. The inset schematics show a circular cell with radius 5 um. The observed distance between tip and start predicts where on the surface the next pseudopod starts. The pseudopod arrows are drawn perpendicular to the curvature, as is observed experimentally.

To identify how the extension of new pseudopodia steers the cell in a gradient we have analyzed the angle of the next pseudopod relative to the current direction of movement of the cell. The data are complex, and therefore we show in Fig. 2 two situations in which the current direction is either towards the cAMP gradient or at an angle of about 90 degrees; the complete data set is presented in supplemental information Fig. S2. In buffer, the next pseudopod is extended at an angle of ∼ 55 degrees to the right or left relative to the current pseudopod, leading to a bi-symmetric distribution of angles (grey bars in Fig. 2). When a cell moves accurately in the cAMP gradient (current angle between −20 and + 20 degrees relative to the cAMP gradient; Fig. 2A), the next pseudopod is not extended at 55 +/− 28 degrees, but at 30 +/− 18 degrees. Thus, both the smaller mean and the smaller variation of angles cause a significant bias of pseudopod extension towards the cAMP gradient, by which the orientation of the cell is preserved. The inset of Fig. 2A reveals that this smaller angle is caused by the fact that the next pseudopod originates closer towards the tip of the previous pseudopod, from 4.39 +/− 0.16 µm for cells in buffer to 2.7 +/− 0.4 µm for cells moving towards cAMP (means and SEM). The second situation, shown in Fig. 2B, summarizes the data for cells that do not move accurately towards cAMP but at an angle between 70 and 110 degrees to the left. The results show that the angle of the next splitting pseudopod is either ∼70 degrees to the left or ∼18 degrees to the right. This directional bias is again due to the altered distance between the tip of the current pseudopod and the start of the new pseudopod. The pseudopod to the left starts further away from the tip at 6.1 µm by which the angle increases to ∼70 degrees, leading to a correction towards cAMP of 70−55 = 15 degrees compared to the extension in buffer. On the other hand, the pseudopod to the right starts nearby the tip at 2.0 µm, thereby is extended ant an angle of 18 degrees, causing a correction towards cAMP of 55−18 = 37 degrees. The complete data set with many different current directions (supplemental figure S2) confirms two key conclusions: First, the bias in direction is caused by the bias in position where the pseudopod emerges; the pseudopod is subsequently extended perpendicular to the local curvature of the membrane. Second, the bias is asymmetric; a turn to the left is caused by a ∼37 degree bias of right pseudopod and ∼15 degree bias of the right pseudopod. Thus, cells steer in a gradient of cAMP by positional/directional bias of the alternating right/left extension of pseudopodia; this bias is maximally 52 +/− 3 degrees per two pseudopodia (see legend figure S2).

### The role of persistence in chemotaxis

**Figure 3:**
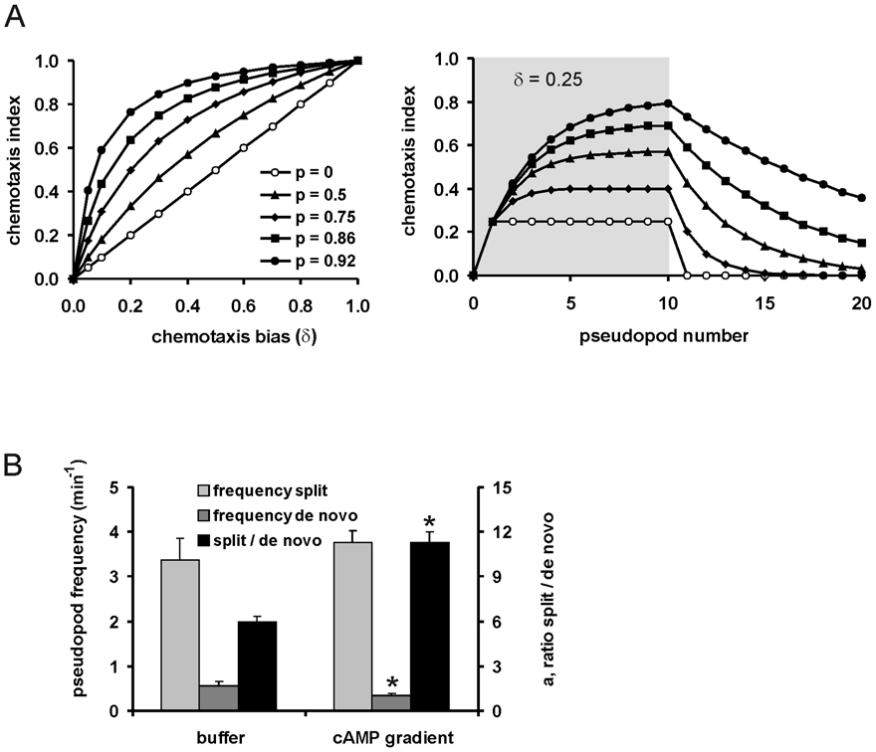
Role of persistence in chemotaxis. A. Theoretical analysis of persistence and chemotaxis bias on chemotactic movement towards the gradient (see supplemental information appendix S1 for equations). In the absence of persistence the chemotactic response is immediate and identical to the chemotactic bias. With persistence the response slowly increases to a higher steady state and persists after removal of the gradient. At the measured [36] threshold for chemotaxis with *δ* = 0.1, the observed persistence of *p* = 0.92 for wild type cells will lead to a ∼5-fold increase of chemotaxis index. B. Effect of a cAMP gradient on the frequency of pseudopod splitting and de novo pseudopodia. Data are means and SEM, n = 28 cells; *, significantly different from buffer at P<0.01. The ratio (*a*) of splitting/de novo pseudopodia is related to the persistence (*p*), according to *p* = *a*/(1+*a*).

We investigated, theoretically and experimentally, how persistence and orientation collaborate to improve chemotaxis (see supplemental information appendix S1 for equations). Assume that cells have persistence *p*, which is the probability to continue movement in the same direction. Also assume that cells exposed to a cAMP gradient have a chemotaxis bias *δ*, which is identical to the chemotaxis index in the absence of persistence. A model for chemotaxis with persistence shows that enhanced persistence will result in an increase of the chemotaxis index, especially in shallow gradients with small values of *δ* (Figure 3A). Moreover, when the chemotactic signal is removed, cells with strong persistence continue to move in the direction of the gradient during a prolonged period of time. Conversely, cells that move in buffer with strong persistence and then exposed to a chemotactic gradient will obtain this high chemotaxis index very slowly.

Previous studies on how cells move in buffer have shown that split pseudopodia are extended predominantly alternating left/right at a small angle leading to a nearly straight persistent path, while *de novo* pseudopodia are extended in nearly random directions pseudopod. Therefore persistence is determined by the ratio (*a*) of split/*de novo* pseudopodia [16]. Pseudopod extension and cell movement was analyzed for 28 cells moving in buffer or exposed to a cAMP gradient. In the absence of cAMP, cells extend ∼3.4 split and ∼0.6 de novo pseudopodia per minute. The split/*de novo* ratio *a* = 6.0 +/− 1.0 (mean and SEM, n = 28). In a shallow gradient of cAMP, the extension of split pseudopodia is not significantly altered, whereas cells extend significantly less de novo pseudopodia, resulting in an enhanced split/*de novo* ratio of *a* = 11.3+/− 2.1 (figure 3B). Thus, cells in a cAMP gradient have a very strong persistence, which amplifies the small bias of pseudopod orientation towards the gradient, and stores this directional movement for prolonged periods of time.

### Major corrections of direction

**Figure 4:**
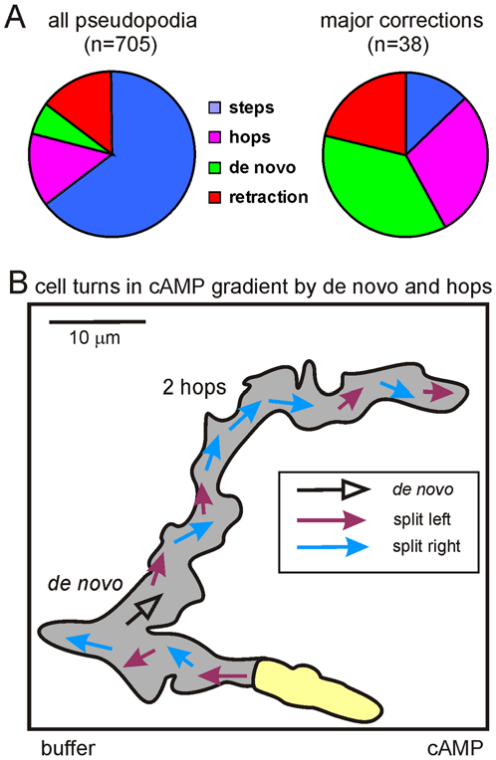
Correction of large deviations from the cAMP gradient. In shallow cAMP gradients cells occasionally move in the wrong direction with an angle >90 degrees relative to the gradient. At some moment these cells make turns in the correct direction. The pseudopodia were characterized that brought the cells back on track. A, the incidence of occurrence demonstrates that major corrections are enriched in hops (consecutive right/right or left/left splitting pseudopodia) and *de novo* pseudopodia. Panel B shows a representative example of a cell that moved ∼180 degrees in the wrong direction. It made a few L/R steps in the wrong direction at ∼180 degrees, a sharp turn by a *de novo* pseudopod, then again a few L/R steps at ∼70 degrees; the cell came on-track by two right hops, and then continued with L/R steps in the direction of the gradient. The grey area shows the surface covered by the cell during this movement.

The aforementioned results suggest that cells moving in a cAMP gradient stay on-track by multiple mechanisms: suppression of random *de novo* pseudopodia (Fig. 3B), selective retraction of poorly oriented pseudopodia (Fig. S1) and adjusting the position and thereby the direction of newly split pseudopodia (Fig. 2 and S2). It should be noted, however, that the direction of pseudopod extensions has a large standard deviation in these shallow gradients (about 20 degrees). Therefore, cells occasionally move in a “very wrong” direction, and we have investigated how such cells reorient in the cAMP gradient. Cells may make major corrections by multiple mechanisms, including a bias of left/right pseudopod splitting steps by which the cells gradually reorient (like novice ice-skaters make a curve), a larger correction through a left/left or right/right hop (like professional speed skaters), a well-oriented de novo pseudopod, or selective retraction. We analyzed 26 cells that moved off-track by more than 90 degrees relative to the gradient, and traced the pseupopod(s) that brought the cell back on-track. The results of Fig. 4A show that major corrections by steps (alternating right/left splitting) are rare compared to the abundance of steps for on-track cell movement. Also selective retraction of pseudopodia is relatively rare. In contrast, hops (consecutive right/right or left/left splitting) and *de novo* pseudopodia are enriched during major directional changes. Figure 4B shows a typical ∼180 degrees correction with one *de novo* pseudopod and two hops.

### Pseudopod formation in chemotactic mutants

**Figure 5:**
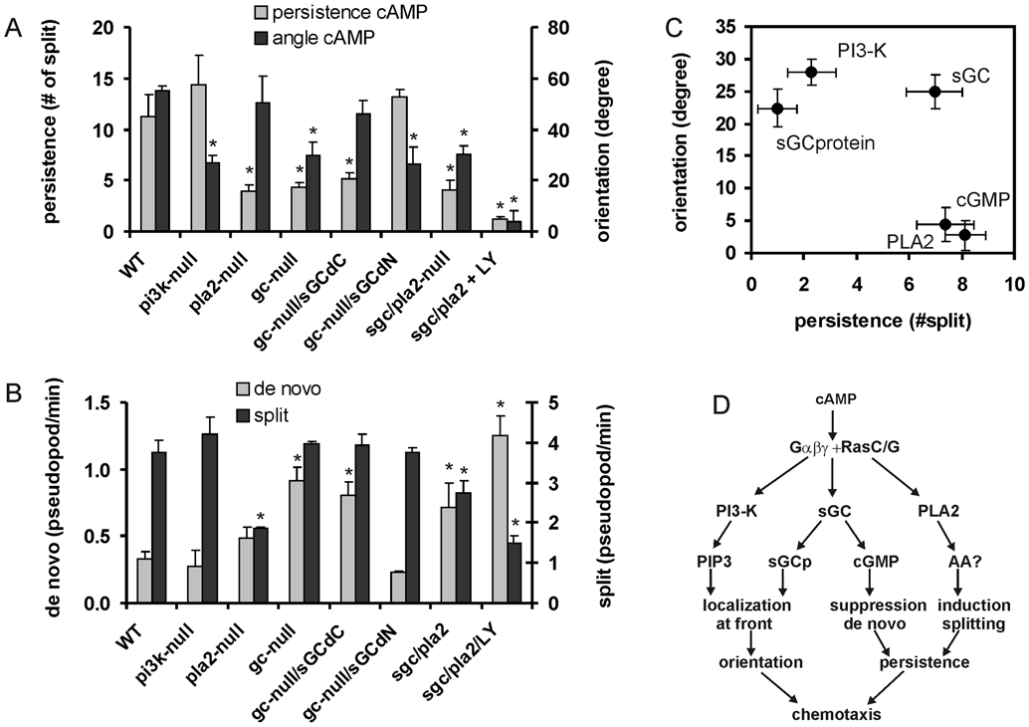
Pseudopod formation in mutants. Mutants with one or multiple mutations were exposed to a shallow cAMP gradient, and analyzed for pseudopod extensions (see table S1 for statistics and additional properties). Panel A shows the persistence and orientation. Persistence is expressed as the number of persistent steps in between two *de novo* pseudopodia, based on the frequencies of split and *de novo* pseudopodia shown in panel B. The orientation is expressed as the maximal correction of splitting pseudopodia as defined in Figure 3C. Data of panels A and B show the means and SEM of 12 wild type cells and 7 or 8 mutant cells, *, significantly different from wild type at P<0.05. C, the contribution of each signaling pathway to persistence and orientation was calculated by taking the difference of two data sets as follows: PI3K, average of difference of WT and *pi3k*-null, and difference between *sgc/pla2*-null and *sgc/pla2*-null + LY; PLA2, difference of WT and *pla2*-null; sGC, difference of WT and *gc*-null; sGCp (sGC-protein), average of difference of *gc*-null and *gc*-null/sGCΔCat, and difference of WT and *gc*-null/sGCΔN; cGMP, average of difference of *gc*-null and *gc*-null/sGCΔN, and difference of WT and *gc*-null/sGCΔCat. D, Model of signaling pathways leading to persistence and orientation. cAMP activates heterotrimeric- and Ras GTP-binding proteins through surface receptors. The activated PI3K and sGC-protein (sGCp) accumulate at the leading edge where they regulate orientation, which is the position and direction in which the pseudopod is extended. The product of sGC, cGMP, suppresses *de novo* pseudopodia predominantly in the rear of the cell, while the product of PLA2, probably arachidonic acid (AA), induces pseudopod splitting; both pathways lead to persistence of pseudopod extension in the direction of previous pseudopodia.

Chemotactic orientation in *Dictyostelium* has been attributed to at least three signaling enzymes, PI3K, PLA2 and guanylyl cyclase [17]. Mutants defective in one or two pathways were exposed to a cAMP gradient. Due to the remaining parallel pathways, the mutants display good chemotaxis albeit slightly diminished compared to wild type cells. We measured persistence as the ratio (*a*) of split/*de novo* pseudopodia, and orientation as the maximal correction of the angle between pseudopodia during splitting (see Fig. S2 for definition). Cells lacking the two most important PI3-kinases exhibit persistence in a cAMP gradient that is essentially identical to that of wild-type cells. However, the orientation of splitting pseudopodia is strongly diminished (Fig. 5A): Wild-type cells can correct the direction of splitting pseudopodia by as much as 52 +/− 3 degrees per two pseudopodia, whereas *pi3k-1/2*-null cells change direction by only 27 +/−3 degrees. Conversely, cells lacking PLA2 activity exhibit excellent orientation, but poor persistence, which is due to the reduced frequency of pseudopod splitting (Fig. 5B). Cells lacking the two known guanylyl cyclases exhibit both poor persistence and orientation. The low persistence of these *gc*-null cells is not due to lower splitting frequency as in *pla2*-null cells, but to the high frequency of *de novo* pseudopodia. The soluble sGC provides nearly all guanylyl cyclase activity of starved *Dictyostelium* cells [18]. Mutation studies suggest that sGC plays two roles during chemotaxis. It functions as protein at the leading edge that may aid orientation, and it acts as enzyme producing cGMP that may suppress *de novo* pseudopodia [16], [19]. Two mutant sGC proteins were expressed in *gc*-null cells. The sGCΔCat can not produce cGMP, but still localizes to the leading edge; these *gc*-null/sGCΔCAT cells still have poor persistence, but exhibit greatly improved orientation. The sGCΔN mutant has the opposite properties: it produces cGMP but does not localize to the leading edge. Interestingly, expression of this protein in *gc*-null cells restores persistence but has no effect on orientation. Finally, we analyzed a mutant that lacks both PLA2 and sGC. In buffer these cells move at a similar rate as wild type cells but show little displacement due to low persistence [16]. In a shallow gradient these *sgc/pla2*-null cells also exhibit strongly reduced but still significant persistence and orientation of pseudopod extensions, resulting in a chemotaxis index of 0.65 +/− 0.02. Upon addition of LY294002, an inhibitor of PI3K and perhaps other signaling pathways such as TorC2 [20], the chemotactic system collapses: the angle between subsequent pseudopodia is no longer affected by the cAMP gradient and persistence becomes extremely defective, leading to a chemotaxis index of 0.01 +/− 0.05.

The contribution of each of the four signaling pathways to persistence and orientation was calculated (Fig. 5C), demonstrating that PI3K and sGC-protein mediate orientation of the cell, while PLA2 and cGMP modulate persistence. PLA2 induces persistence by enhancing pseudopod splitting, while cGMP suppresses the formation of *de novo* pseudopodia. PI3K/PIP3 and sGC-protein accumulate at the side of the cell closest to the cAMP source, where they are components of an F-actin inducing control loop [17], [19], [21], [22].

### Persistence and orientation during chemotaxis in natural gradients

**Figure 6:**
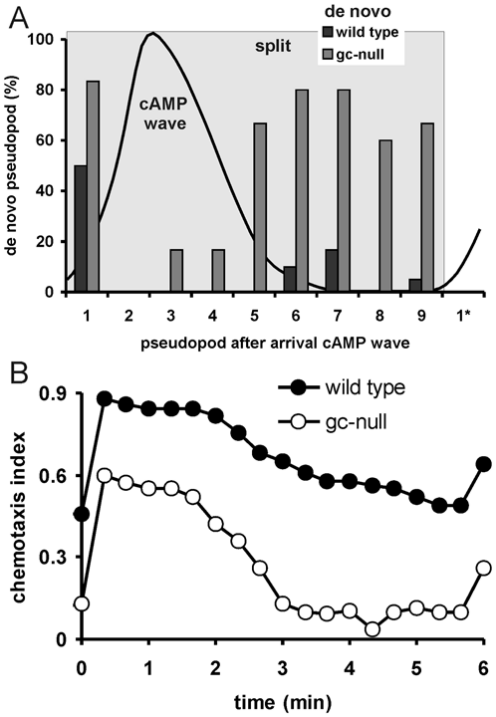
Pseudopod formation and chemotaxis in natural gradients. A, pseudopod formation during a natural wave. The cAMP wave was calculated from published data [23], [37]. The sharp increase of cell speed at the start of the wave [23], [24] was used to align the cAMP wave with present observations. Cells extend split and *de novo* pseudopodia; indicated are *de novo* pseudopodia as fraction of all pseudopodia. B, the chemotaxis index during a cAMP wave. The data are obtained from 20 cells during 4 late waves, obtained from three independent movies [19].

The chemotactic system of *Dictyostelium* cells is dedicated for cell aggregation towards cAMP that is secreted by the cells with a periodicity of 5 minutes. During cell aggregation, cells are exposed to waves of cAMP that increase in concentration and point in the direction of the aggregation centre during about 90 s, then decline and point in the opposite direction during 90 s, whereas cAMP is absent during the remaining 120 s. We recorded movies of aggregating wild type cells and analyzed the movement with Quimp3. The onset of the cAMP waves was deduced from the observed sharp increased of cell speed when the cAMP wave arrives at the cell [23], [24]. The first pseudopod that is extended by a wild type cell after being exposed to the cAMP wave is in 45% of the cells a *de novo* pseudopod and in 55% of the cells a split pseudopod (Fig. 6A). In both cases the pseudopod is oriented rather precisely (the mean angle between pseudopod and aggregation centre is ∼20 degrees). The direction of movement just before extending this first pseudopod deviated 103 degrees from the direction of the aggregation centre for cells that subsequently protrude a *de novo* pseudopod, and 35 degrees if a split pseudopod was extended. In other words, when a cell already moves in the direction of the upcoming chemoattractant gradient, the cell continues its movement by pseudopod splitting, but when the movement is not in the direction of the upcoming wave, the cAMP gradient induces a well-oriented *de novo* pseudopod. After this first pseudopod, nearly all subsequent extensions during the cAMP wave are split pseudopodia. Interestingly, after the cAMP wave has passed wild-type cells, *de novo* pseudopodia are still suppressed, and >95% of all pseudopodia are formed by splitting. Consequently, cells continue their movement in the direction of the aggregation centre, as represented by the chemotaxis index that declines only slowly after the cAMP wave has passed the cells (Fig. 6B). Due to hops and steps that are not exactly 55 degrees, cells gradually lose orientation relative to the position of the aggregation centre, by which at the end of this 2 min gradient-less period the chemotaxis index has declined and a substantial fraction of the cells move off-track. When the new wave arrives, the on-track cells continue to extend split pseudopodia, while the direction of off-track cells is corrected by extending a *de novo* pseudopod.

The role of persistence and de novo pseudopodia in chemotaxis during natural aggregation was investigated using a mutant that lacks the two guanylyl cyclases. These *gc*-null cells are defective in suppression of *de novo* pseudopodia (Fig. 5B) and exhibit a very strong phenotype during natural aggregation [19]. During the cAMP wave, *gc*-null cells as wild type cells extend split pseudopodia in the direction of the aggregation centre, leading to significant chemotaxis (Fig. 6). However, after the cAMP wave has passed by, *gc*-null cells immediately extend many *de novo* pseudopodia. As a consequence, the chemotaxis index drops immediately and nearly all cells are off-track when the next cAMP wave arrives at the cells, which therefore induces many de novo pseudopodia to correct the direction of movement.

The experimental observations are in close agreement with model predictions (Figs 6 and 3A): First, wild-type cells with strong persistence (p = 0.92) retain chemotaxis after the cAMP gradient has disappeared. Second, *gc*-null cells with reduced persistence (p = 0.75) have a lower chemotaxis index and rapidly lose chemotaxis after removal of the signal. Third, theory predicts a trade-off for improved chemotaxis by strong persistence, which is slow re-orientation to a new chemotactic signal. In natural waves, *Dictyostelium* cells circumvent the trade-off by extending a *de novo* pseudopod to immediately move in the correct direction of the new gradient and only then use persistence by pseudopod splitting to stay on-track.

## Discussion

**Figure 7:**
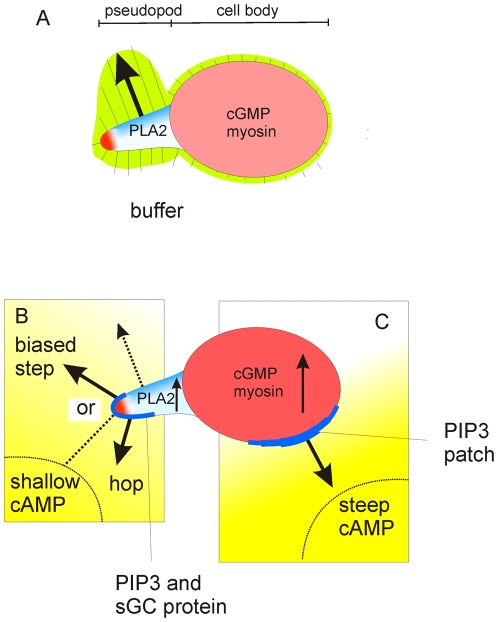
Model for cell movement and chemotaxis using persistence and orientation. Panel A shows a cell in buffer that has made a split to the left. The line segments indicate the probability (in % per µm circumference) that a pseudopod will emerge at that position; the direction of these line segments is perpendicular to the surface. Inhibitors in red may explain the observed low frequency of pseudopodia in the cell body (cGMP) and at the tip (unknown), while activators in blue may explain the high probability of pseudopod formation in the present pseudopod (PLA2) at the right side (unknown). In a cAMP gradient (panels B and C), cGMP and PLA2 are activated which causes global inhibition of pseudopodia in the cell body and enhanced pseudopod formation in the pseudopod, leading to enhanced persistence. Other signaling molecules, such as PIP3 and sGC protein but probably more, accumulate locally at the side of the cell closest to the gradient. In conjunction with the endogenous activators and inhibitors, these gradient-induced activators establish the position where a pseudopod emerges. In buffer the cell would extend a right pseudopod (dotted black arrow). The shallow gradient (panel B) may bias the position closer to the tip yielding either a better oriented right step, or a left hop; a new pseudopod is extended rarely at the present tip because of the endogenous inhibitor. The steep cAMP gradient (panel C) induces a very strong PIP3 patch that nearly always induces a pseudopod at that position, even when it occurs in the cell body or at the very tip of a pseudopod.

Many eukaryotic cells extend pseudopodia. It appears that the movement of *Dictyostelium* cells in a chemotactic gradient is firmly based on the ordered extension of pseudopodia in the absence of external cues [16] (Figure 7). Pseudopodia are extended always perpendicular to the surface curvature, independent of the direction of the gradient. Therefore the direction of movement depends on the position where a pseudopod emerges and on the local curvature of the membrane. Two types of pseudopodia have been recognized, splitting of the current pseudopod, and a pseudopod formed de novo at the cell body [15]. In buffer, pseudopod splitting is highly coordinated with a strong alternating right/left bias. Since pseudopodia are formed nearby the parental pseudopod, they are extended at a small angle relative to each other, resulting in a relatively straight zigzag trajectory. In contrast, de novo pseudopodia are extended at nearly random positions on the cell body, and therefore in any direction. Mutant analysis revealed that cGMP, through the formation of myosin filaments, suppresses the formation of de novo pseudopodia, whereas PLA2 signaling, by unknown mechanisms, induces pseudopod splitting. The length of the persistent zigzag path depends on the ratio of splitting/de novo pseudopodia, and therefore on cGMP and PLA2 activity.

The cAMP gradient induces a bias in the direction of the pseudopodia towards the gradient (defined as orientation). The pseudopod emerges more closely towards the gradient side of the cell, and is then extended perpendicular to the local curvature of the membrane in the direction of the gradient. We observed minimal effects of the cAMP gradient on the size, frequency and bending of pseudopod extension. In the few cases that a cell extends multiple pseudopodia and one is retracted, it appears that the pseudopod with the best orientation relative to the gradient is maintained. Therefore, the strategy for chemotaxis is to extend or maintain pseudopodia at the side of the cell closest to the chemotactic gradient. At that side the surface curvature of the cell is approximately at a right angle relative to the gradient, by which pseudopodia perpendicular to this surface are extended automatically towards the attractant.

The position where a pseudopod emerges is likely determined by local and global activators and inhibitors. We have investigated how four signaling molecules contribute to chemotaxis. Stimulated PLA2 and cGMP enhance splitting and suppress de novo pseudopodia, respectively, and thereby enhance persistence, but have no effect on the orientation of the pseudopodia. In contrast, the sGC protein and PIP3 signaling do not affect splitting frequency and persistence, but strongly influence the position where a new pseudopod emerges. We propose that the pseudopod stimulatory activity of sGC protein and PIP3 will combine with endogenous activators and inhibitors, thereby inducing a shift of the position where the pseudopod emerges. In a shallow cAMP gradient, sGC protein and PIP3 weakly accumulate at the side of the cell closer to the gradient [25] (Veltman, Bosgraaf and Van Haastert, unpublished data). This weak positional cue in a shallow gradient may induce a bias of pseudopod relatively easy in the activating environment of the splitting pseudopod, but more difficult in the cell body. We have also analyzed how pseudopodia are extended in a steep cAMP gradient that occur during natural cell aggregation (Fig. 6) or in gradients with different steepness (delivered by micropipettes; unpublished data). We observed two phenomena. First, cells that happen to move already towards the exposed gradient continue with biased pseudopod splitting in the direction of the gradient, whereas cells that moved in other directions extend a de novo pseudopod in the direction of the new gradient. Second, very shallow cAMP gradients induce a directional bias of splitting pseudopodia (half-maximal effect at a gradient of 0.2 nM/µm), while ten-fold steeper gradients are required to induce a de novo pseudopodia in the direction of the gradient (unpublished data). It has been shown that in a steep gradient, the sGC protein and PIP3 strongly accumulate at the membrane [19], [25], [26], and may bias the position where pseudopodia emerge more strongly than in a shallow gradient: steep gradients can induce splitting near the tip of the present pseudopod in cells on-track, but can also induce a well-oriented de novo pseudopod in the cell body to re-orient the cell in the gradient.

The main conclusion of this study is that the time and position of pseudopodia formation is the result of integration of endogenous and gradient-induced activating signals. This view on pseudopod formation may help to explain large differences in the motility behavior between cells. Many cells are polarized, which means that cells have one (sometimes multiple) polarity axis of biochemical, structural and/or functional heterogeneity. Feeding *Dictyostelium* cells, or cells starved for a few hours, have a very plastic polarity. Such cells continuously change directions, and chemotactic stimulation at the current rear of the cell often induces a new front at that position, by which the cell reverses direction [27]. Cells starved for ∼5–7 hours obtain a more permanent polarity axis, pseudopodia appear nearly exclusively at the current front, even when cells receive strong chemotactic stimulation at the current rear; those cells do not reverse direction but make a U-turn [27], [28]. This polarity of pseudopod extension is most likely related to the strong suppression of de novo pseudopodia in the rear and cell body. Indeed, it has been shown that the transition of flexible polarity to the more rigid polarity around 5–7 hours of starvation in *Dictyostelium* is due to the cGMP-signaling pathway that suppresses de novo pseudopodia [17]. In cells with flexible polarity, pseudopodia are easily induced at any position of the cell, and a strong gradient may induce a well-oriented pseudopod, such as in compass models for chemotaxis [29]. In strongly polarized cells, however, pseudopod formation occurs preferentially at the front, and the bias of direction by the gradient is then restricted to relatively small changes of direction, such as proposed in the local coupling model for chemotaxis [30].

In *Dictyostelium*, a gradient of cAMP, compared to buffer, has little effect on many properties of pseudopodia cells, such as frequency and size of pseudopodia. Therefore, a cAMP gradient does not strongly interfere with the intrinsic pseudopod cycle; the gradient does not induce a new pseudopod, but produces a bias in the probability where the next pseudopod will emerge. Chemotaxis in *Dictyostelium* appears, therefore, pseudopod-based/gradient-biased. In contrast, neutrophils in the absence of chemoattractant are nearly immobile. A uniform stimulus of chemoattractant induces the extension of pseudopodia in random directions, which can be position-biased in a gradient of attractant [31]–[33]. Thus chemotaxis in neutrophils appears gradient-induced. This large differences between *Dictyostelium* and neutrophils in chemotactic appearance may have a common basis, which is the relative presence of pseudopod inducers and inhibitors. Neutrophils in buffer may have very low pseudopod-activating activity that is silenced by strong uniform inhibition; in combination with strong gradient-induced local activators this leads to gradient-induced pseudopodia. Compared to neutrophils, *Dictyostelium* cells in buffer may have more pseudopod-inducing activity and lower uniform inhibition leading to a strong cycle of pseudopod activity; the gradient induces just a small bias of the position where the inevitable next pseudopod will appear.

Summarizing, the analysis of discrete pseudopod events in buffer and shallow gradients has provided fundamental insight how cells employ pseudopod splitting and *de novo* pseudopod extension for persistence and orientation. Cell movement in buffer and in chemotactic gradients is dominated by the position where pseudopodia emerge. Local signaling molecules that are induced by the gradient integrate with endogenous signaling molecules for ordered pseudopod extension, thereby inducing a bias of the position at the cell boundary where a pseudopod emerges. By self-organization, the pseudopod then extends perpendicular to the surface for ∼12 seconds, growing to a size of ∼5 µm. Upon flow of cytoplasm into the pseudopod, and retraction of the rear, the cell has moved in the direction of the gradient.

## Methods

The strains used are wild type AX3, *pi3k*-null strain GMP1 with a deletion of *pi3k1* and *pi3k2* genes [34], *pla2*-null with a deletion of the *plaA* gene [35], *sgc/gca*-null cells (abbreviated as *gc*-null cells) with a deletion of *gca* and *sgc* genes, *gc*-null/sGCΔCat expressing sGC-D1106A in *gc*-null cells, *gc*-null/sGCΔN expressing sGC with the N-terminal deletion of 877 amino acids in *gc*-null cells [19], and *sgc/pla2*-null cells with a deletion of *sgc* and *pla2A* genes [17]. Cells were grown in HG5 medium (contains per liter: 14.3 g oxoid peptone, 7.15 g bacto yeast extract, 1.36 g Na_2_HPO_4_⋅12H_2_O, 0.49 g KH_2_PO_4_, 10.0 g glucose), harvested in PB (10 mM KH2PO4/Na2HPO4, pH 6.5), and allowed to develop in 1 ml PB in a well of a 6-wells plate (Nunc) till they formed aggregation territories. Cells lacking PI3K do not develop well on a solid support, and were starved in suspension with cAMP pulses (100 nM cAMP applied every 6 minutes between 2 and 5 hours of starvation). Chemotaxis competent cells were exposed to a cAMP gradient in a modified Zigmond chamber with 1 mM cAMP in the source agar block and a bridge of 2 mm [19]. Cells were observed ∼700 µm from the source for 15 minutes starting at ∼10 minutes after the start of the gradient. At these conditions a stable spatial cAMP gradient is established with an absolute spatial gradient of 0.5 nM/µm, a relative gradient of 0.7% concentration difference across the cell, and a mean concentration of 650 nM cAMP. Movies were recorded with an inverted light microscope (Olympus Type CK40 with 20× objective) and images were captured at a rate of 1 frame/second with a JVC CCD camera.

Images were analyzed with the automatic pseudopod-tracking algorithm Quimp3, which is described in detail in [14]. In short, the phase contrast movie was converted to a black and white movie using the “phase contrast to BW” macro that is included in the Quimp3 package. Some manual adjustment was required to close a few gaps in the cell silhouette. The resulting file was used as input file for the Quimp3 analysis. The pseudopodia were detected using the default parameters of the macro. The automated pseudopod tracking method identifies the position where a pseudopod starts its extension and the position where the tip of the pseudopod stops growing. The output file contains the frame number and x,y-coordinates of these positions, which were used in Excel to perform the calculations on pseudopod size, interval, direction to gradient, etc. The automated algorithm also annotates each pseudopod as de novo versus splitting (with assignment of the parental pseudopod from which it was split).

The aim of this study is to investigate pseudopod extension during chemotaxis. Since potential defects of pseudopod behavior in mutants may be due to the reduced chemotaxis of the mutants, we only analyzed cells that have a chemotaxis index of 0.6–0.7 (see Table S1 in supplemental information, presenting additional pseudopod properties of the mutants). A typical database contains information from 200–300 pseudopodia obtained from 6–10 cells from two independent movies. We collected one large database for wild type cells containing 835 pseudopodia from 28 cells in 4 independent movies, and typical databases for each mutant. The data are presented as the means and standard deviation (SD), or standard error of the means (SEM) where n represents the number of pseudopodia or number of cells analyzed, as indicated.

## Supporting Information

Figure S1 Orientation of Dictyostelium cells in shallow gradients by selective retraction of existing pseudopodia. When cells have two active pseudopodia, at some moment one pseudopod will be retracted. The figure shows the angle relative to the gradient of the retracted and maintained pseudopod as means and SEM (n = 50).
(0.03 MB PDF)

Figure S2 Orientation of Dictyostelium cells in shallow gradients by a bias of extending pseudopodia. The predominant way Dictyostelium cells move is by splitting-off pseudopodia alternating to the right and left. To identify how cells steer in a gradient with alternating pseudopodia we have to separate between right/left and left/right; here we present the data on the next split to the right after the previous split to the left. A, schematic of analysis. Longer series of alternating right/left splitting pseudopodia were analyzed for the angle of the present pseudopod towards the gradient, the angle of the present pseudopod towards the next pseudopod, and the distance d between tip of present and start of next pseudopod. The red arrow in the cell outline indicates the present split pseudopod to the left, the blue arrow outside the cell indicates the next split pseudopod to the right. Data are means and SEM with in total 283 pseudopodia. Panels B and C presents the distance d or angle respectively. The data were determined for the present pseudopod to the left, relative to the next pseudopod to the right, and are presented as a function of the direction of the present pseudopod towards the cAMP gradient. Orientation is defined in panel C as the difference between the three bars at the right and left, respectively. Schematics D show geometry of pseudopodia in buffer and three situations with different of the present pseudopod to the cAMP gradient; the dotted black arrow indicates the position where a pseudopod would be extended in buffer. The gradient modulates the distance d and thereby the angle, such that pseudopodia become better oriented towards the gradient.
(0.15 MB PDF)

Table S1 Pseudopod properties of Dictyostelium mutants in a shallow cAMP gradient. Wild type AX3 cells (WT) and mutant cells were exposed to a shallow cAMP gradient, movies were recorded and pseudopod extensions were analyzed. Data were obtained from two movies for each mutant strain, with a wild type recorded in parallel. n is the number of experiments; two values are given, the number of cells and the number of pseudopodia, respectively. Additional movies were recorded for wild type cells to obtain a larger data set of 835 pseudopodia extended by 28 cells for detailed analysis (see manuscript). Cells selected for pseudopod analysis have a chemotaxis index between 0.6 and 0.7 to exclude pseudopod behavior due to differences in chemotaxis index between strains (with the exception of sgc/pla2-null cells with LY294002, which have poor chemotaxis). The mean chemotaxis index of all cells in the field is∼0.8 for WT, pi3k-null and pla2-null cells, ∼0.75 for sgc/pla2-null cells, ∼0.65 for gc-null/sGCdeltaC and gc-null/sGCdeltaN cells, and ∼0.6 for gc-null cells.
(0.03 MB PDF)

Appendix S1 Equation of chemotaxis index for cells with a chemotaxis bias and persistence of movement.
(0.07 MB PDF)
