# Supplementary figures and images for: Navigation of Chemotactic Cells by Parallel Signaling to Pseudopod Persistence and Orientation
Source: PLoS One. 2009 Aug 31;4(8):e6842. doi: 10.1371/journal.pone.0006842 (PMC2729408; doi:10.1371/journal.pone.0006842)

Figure S1.

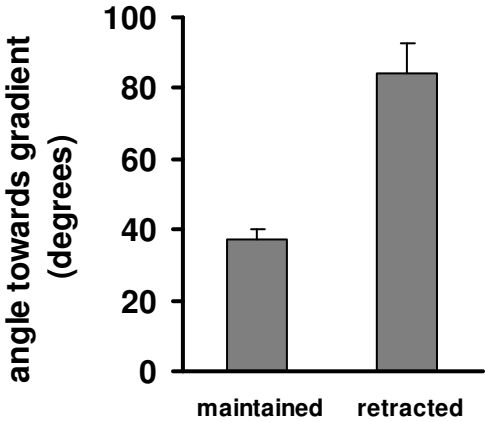

Supplement: Figure S1 — Orientation of Dictyostelium cells in shallow gradients by selective retraction of existing pseudopodia. When cells have two active pseudopodia, at some moment one pseudopod will be retracted. The figure shows the angle relative to the gradient of the retracted and maintained pseudopod as means and SEM (n = 50). (0.03 MB PDF) [file pone.0006842.s001.pdf]

Figure S2.

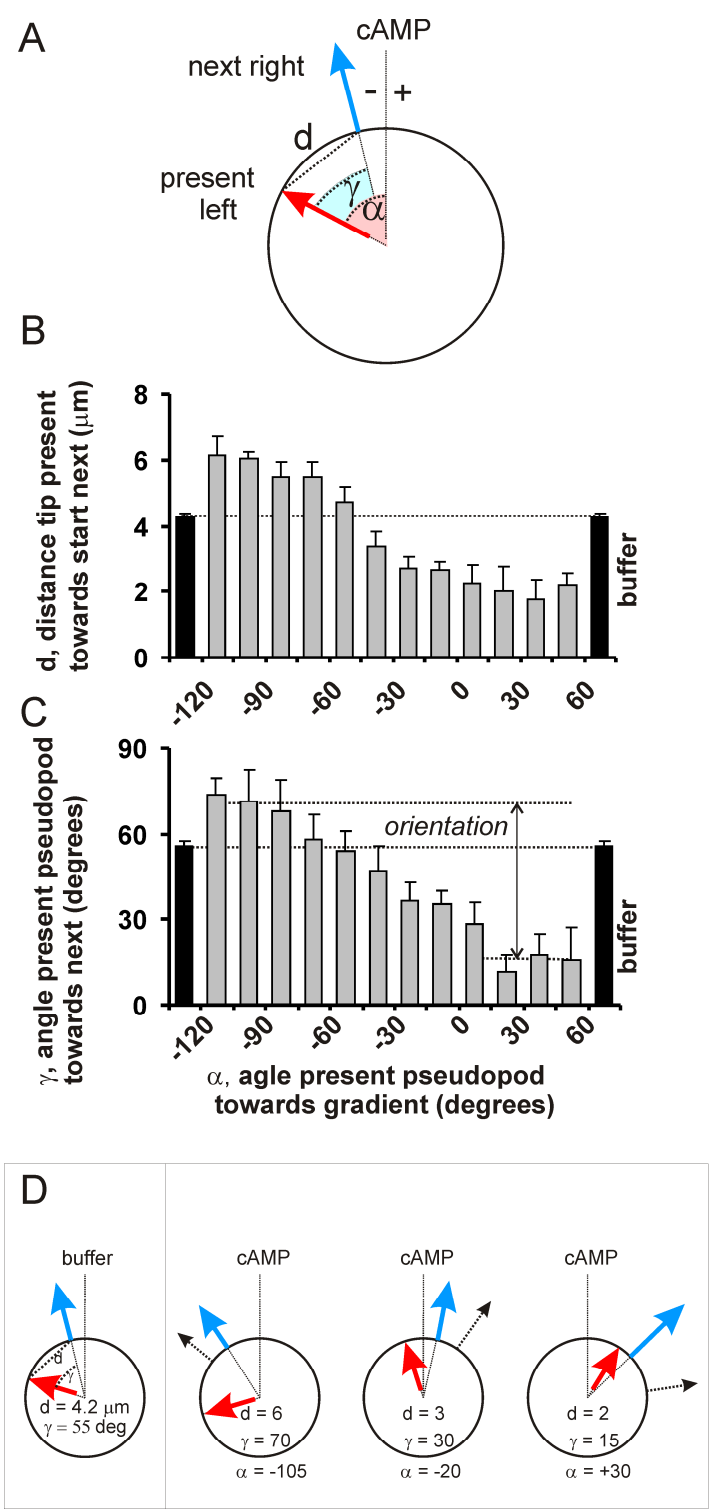

Supplement: Figure S2 — Orientation of Dictyostelium cells in shallow gradients by a bias of extending pseudopodia. The predominant way Dictyostelium cells move is by splitting-off pseudopodia alternating to the right and left. To identify how cells steer in a gradient with alternating pseudopodia we have to separate between right/left and left/right; here we present the data on the next split to the right after the previous split to the left. A, schematic of analysis. Longer series of alternating right/left splitting pseudopodia were analyzed for the angle of the present pseudopod towards the gradient, the angle of the present pseudopod towards the next pseudopod, and the distance d between tip of present and start of next pseudopod. The red arrow in the cell outline indicates the present split pseudopod to the left, the blue arrow outside the cell indicates the next split pseudopod to the right. Data are means and SEM with in total 283 pseudopodia. Panels B and C presents the distance d or angle respectively. The data were determined for the present pseudopod to the left, relative to the next pseudopod to the right, and are presented as a function of the direction of the present pseudopod towards the cAMP gradient. Orientation is defined in panel C as the difference between the three bars at the right and left, respectively. Schematics D show geometry of pseudopodia in buffer and three situations with different of the present pseudopod to the cAMP gradient; the dotted black arrow indicates the position where a pseudopod would be extended in buffer. The gradient modulates the distance d and thereby the angle, such that pseudopodia become better oriented towards the gradient. (0.15 MB PDF) [file pone.0006842.s002.pdf]
