# Supplementary material for: Navigation of Chemotactic Cells by Parallel Signaling to Pseudopod Persistence and Orientation
Source: PLoS One. 2009 Aug 31;4(8):e6842. doi: 10.1371/journal.pone.0006842 (PMC2729408; doi:10.1371/journal.pone.0006842)
Supplement: Table S1 — Pseudopod properties of Dictyostelium mutants in a shallow cAMP gradient. Wild type AX3 cells (WT) and mutant cells were exposed to a shallow cAMP gradient, movies were recorded and pseudopod extensions were analyzed. Data were obtained from two movies for each mutant strain, with a wild type recorded in parallel. n is the number of experiments; two values are given, the number of cells and the number of pseudopodia, respectively. Additional movies were recorded for wild type cells to obtain a larger data set of 835 pseudopodia extended by 28 cells for detailed analysis (see manuscript). Cells selected for pseudopod analysis have a chemotaxis index between 0.6 and 0.7 to exclude pseudopod behavior due to differences in chemotaxis index between strains (with the exception of sgc/pla2-null cells with LY294002, which have poor chemotaxis). The mean chemotaxis index of all cells in the field is∼0.8 for WT, pi3k-null and pla2-null cells, ∼0.75 for sgc/pla2-null cells, ∼0.65 for gc-null/sGCdeltaC and gc-null/sGCdeltaN cells, and ∼0.6 for gc-null cells. (0.03 MB PDF) [file pone.0006842.s003.pdf]

Table S1 Pseudopod properties of *Dictyostelium* mutants in a shallow cAMP gradient

Strain	n	Chemotaxis index		Pseudopod size (µm)		Pseudopod Growth time (s)		Pseudopod growth speed (µm/min)		pseudopod interval (s)		Freq split (1/s)		Freq de novo (1/s)		Persistence a, # split pseudopodia		orientation (degrees)	
		mean	SEM	mean	SEM	Mean	SEM	Mean	SEM	mean	SEM	mean	SEM	mean	SEM	mean	SEM	mean	SEM
WT	12/335	0.62	0.03	5.78	0.43	12.23	1.17	0.52	0.02	14.35	2.37	3.76	0.28	0.33	0.05	11.30	2.07	54.87	2.29
pi3k-null	8/227	0.61	0.02	6.66	0.39	12.34	1.27	0.73	0.13	14.13	0.95	4.21	0.43	0.27	0.12	14.34	2.87	26.97	2.70
pla2-null	8/260	0.63	0.02	8.22	0.42	15.04	1.00	0.61	0.02	15.76	0.41	1.85	0.02	0.48	0.09	5.50	0.28	47.47	5.49
gc-null	7/273	0.65	0.01	4.71	0.08	13.11	0.84	0.40	0.02	13.59	0.88	3.96	0.08	0.91	0.10	4.35	0.48	29.92	4.84
gc-null/sGCΔC	8/284	0.64	0.06	4.90	0.15	11.42	1.22	0.49	0.03	13.07	0.85	3.92	0.30	0.80	0.10	5.18	0.55	45.87	5.35
gc-null/sGCΔN	7/193	0.64	0.05	5.43	0.51	13.43	1.11	0.44	0.04	16.28	0.51	3.74	0.15	0.23	0.01	13.15	0.77	26.49	6.57
sgc/pla2-null	7/235	0.65	0.02	4.91	0.37	12.74	0.87	0.44	0.05	16.26	0.43	2.75	0.32	0.71	0.19	4.06	1.01	30.00	3.59
sgc/pla2-null + LY	8/199	-0.01	0.06	5.06	0.18	19.88	3.26	0.33	0.04	24.89	2.90	1.49	0.18	1.25	0.15	1.20	0.20	4.00	3.98
