# Supplementary material for: Navigation of Chemotactic Cells by Parallel Signaling to Pseudopod Persistence and Orientation
Source: PLoS One. 2009 Aug 31;4(8):e6842. doi: 10.1371/journal.pone.0006842 (PMC2729408; doi:10.1371/journal.pone.0006842)
Supplement: Appendix S1 — Equation of chemotaxis index for cells with a chemotaxis bias and persistence of movement. (0.07 MB PDF) [file pone.0006842.s004.pdf]

Appendix S1. Equation of chemotaxis index for cells with a chemotaxis bias and persistence of movement.

The chemotaxis index Ψ is the fraction of cells moving towards cAMP. A cell moving in buffer exhibits persistence p, which is the probability to continue movement in the same direction. In a gradient of cAMP the cell also exhibits a chemotactic bias δ, which is defined as the probability to move towards the cAMP source (δ is identical to the chemotaxis index Ψ when $p = 0$).

Chemotaxis index

With these definitions, $\Psi(n+1)$ is the fraction of cells moving to cAMP at the $(n+1)^{th}$ pseudopod, and equals the fraction of cells moving to cAMP at the nth pseudopod that continue to move in that direction due to persistence [$\Psi(n) * p$], plus the fraction that did not persist in the direction of the gradient but will move to cAMP due to a chemotactic bias [$\Psi(n)*(1-p)*\delta$], plus the fraction that did not move to cAMP but will move to cAMP due to a chemotactic bias [$(1- \Psi(n))*\delta$].

$$\Psi(n+1) = \Psi(n)p + \Psi(n)(1-p)\delta + [1-\Psi(n)]\delta \quad (1)$$

Since pseudopod extension is a discrete event, this equation was not solved by integration, but used numerically to calculate the chemotaxis index after the $(n+1)^{th}$ pseudopod.

At equilibrium $\Psi(n+1) = \Psi(n)$, yielding

$$\Psi(\propto) = \frac{\delta}{1-p+p\delta} \quad (2)$$

Kinetics

To find an equation that expresses the number of pseudopodia at which half-maximal equilibrium is reached, equation (1) was written as a differential equation

$$d\Psi(n)/dn = -\Psi(n)[1-p+p\delta] + \delta \quad (3)$$

after integration this yields

$$\Psi(n) = \Psi(\propto) - [\Psi(\propto) - \Psi(0)]e^{-n(1-p+p\delta)} \quad (4)$$

where $\Psi(\propto) = \dfrac{\delta}{1-p+p\delta}$ is the chemotaxis index at equilibrium. (5)

and $\Psi(0)$ is the chemotaxis index at n = 0 (6)

Half-maximal equilibrium is reached at $n_{0.5} \approx \ln 2/(1-p+p\delta)$ (7)

Again, since pseudopod extension is a discrete event, $n_{0.5}$ must be a natural number, indicating that $n_{0.5}$ scales with $1/(1-p+p\delta)$.

Persistence

The cellular basis for persistence is the splitting pseudopod, which occurs at a small angle and frequently alternating to the right and left leading to a persistent zig-zag trajectory. This persistent path is interrupted by a de novo pseudopod in a random direction (Bosgraaf and van Haastert, 2009).

Therefore, $p = \frac{S}{S+N} = \frac{a}{1+a}$, (8)

where S and N are the frequencies of pseudopod splitting and de novo, respectively, and *a* is the number of split pseudopods in between two de novo pseudopodia.

Bosgraaf, L., and van Haastert, P. J. M. (2009). The Ordered Extension of Pseudopodia by Amoeboid Cell in the Absence of external Cues. PLoS ONE 4: e5253.
